# A uniform human multimodal dataset for emotion perception and judgment

**DOI:** 10.1038/s41597-023-02693-z

**Published:** 2023-11-07

**Authors:** Sai Sun, Runnan Cao, Ueli Rutishauser, Rongjun Yu, Shuo Wang

**Affiliations:** 1 Frontier Research Institute for Interdisciplinary Sciences, Tohoku University, Sendai, 980-8578 Japan; 2 Research Institute of Electrical Communication, Tohoku University, Sendai, 980-8577 Japan; 3 Department of Radiology, Washington University in St. Louis, St. Louis, MO 63110 USA; 4 Departments of Neurosurgery and Neurology, Cedars-Sinai Medical Center, Los Angeles, 90048 California USA; 5 Department of Management, Marketing, and Information Systems, Hong Kong Baptist University, Hong Kong, China

**Keywords:** Cognitive neuroscience, Social neuroscience

## Abstract

Face perception is a fundamental aspect of human social interaction, yet most research on this topic has focused on single modalities and specific aspects of face perception. Here, we present a comprehensive multimodal dataset for examining facial emotion perception and judgment. This dataset includes EEG data from 97 unique neurotypical participants across 8 experiments, fMRI data from 19 neurotypical participants, single-neuron data from 16 neurosurgical patients (22 sessions), eye tracking data from 24 neurotypical participants, behavioral and eye tracking data from 18 participants with ASD and 15 matched controls, and behavioral data from 3 rare patients with focal bilateral amygdala lesions. Notably, participants from all modalities performed the same task. Overall, this multimodal dataset provides a comprehensive exploration of facial emotion perception, emphasizing the importance of integrating multiple modalities to gain a holistic understanding of this complex cognitive process. This dataset serves as a key missing link between human neuroimaging and neurophysiology literature, and facilitates the study of neuropsychiatric populations.

## Background & Summary

Faces are among the most ubiquitous social stimuli that people see in everyday life. Although there is a large literature studying face perception in humans, most of these studies focus on a single aspect of face perception using unimodal approaches. To comprehensively understand face perception, we highlighted the importance of multimodal cognitive neuroscience approaches for studying face perception^1^. For example, the response to fearful faces has been comprehensively delineated using a combination of neuronal firing, local field potentials, and hemodynamic activity in the human amygdala^2^. Here, we present a multimodal dataset for studying emotion perception and judgment. A key strength of our dataset is that all participants performed the same emotion judgment task (i.e., a two-alternative forced-choice task) with a gradient of morphed faces along the fear-happy dimension. We have acquired the following behavioral and neural data:

1. Electroencephalogram (EEG): Data from 97 unique neurotypical participants were acquired through a series of 8 experiments. An example result is the late positive potential (LPP), which encodes facial emotion ambiguity^3^. Through a series of experiments^3,4^, we have characterized the LPP in detail and shown that it is specifically associated with behavioral judgments about choices that are ambiguous. This result is consistent with the literature showing that the LPP is sensitive to various types of ambiguity, including ambiguous facial expressions^5^, racially ambiguous faces^6^, socially relevant concepts^7^, affective pictures^8–10^, and stimulus uncertainty^11^.
2. Functional magnetic resonance imaging (fMRI): Data from 19 neurotypical participants were acquired. Example results include activation of the left amygdala for the degree of fearfulness/happiness in the face^12^, and activation of the right amygdala^12^, dorsomedial prefrontal cortex (dmPFC), and ventromedial prefrontal cortex (vmPFC)^3^ for categorical ambiguity of the stimuli. These results are consistent with the role of the amygdala in emotion processing^13–15^, especially fear processing^16–18^, as well as its role in detecting ambiguous stimuli and modulating vigilance and attention accordingly^19–21^. Furthermore, although the anterior cingulate cortex (ACC) has functional segregations (see^22,23^ for details), most of its functions involve processing ambiguity in some form, which requires conflict resolution, ongoing action monitoring, dynamic adjustments in cognitive control, and inversely correlates with confidence in judgment^22,24–30^ (see also^31^ and^32^ for direct results with ambiguous stimuli), consistent with our results.
3. Single-neuron recordings: We had the unique opportunity to record single-neuron activity directly from the human brain in neurosurgical patients undergoing monitoring to localize their epileptic seizures^33^. By recording from 16 neurosurgical patients with depth electrodes (22 sessions in total), we have shown that neurons in the amygdala parametrically encode the degree of fearfulness/happiness in the face and the categorical ambiguity of the stimuli^12^, and neurons in the dmPFC encode the categorical ambiguity of the stimuli^34^. These results are consistent with the above fMRI results.
4. Eye tracking: We acquired eye tracking data from 24 neurotypical participants. Fixations can be compared between task conditions (e.g., ambiguity level^4^ and face size^35^); and facial regions of interest (ROIs) have been delineated. Furthermore, we can compare eye movement in people with autism spectrum disorders (ASD) vs. controls (see below).
5. Autism spectrum disorder (ASD): We acquired behavioral and eye tracking data from 18 participants with ASD and 15 matched controls. We have shown reduced specificity in emotion judgment in participants with ASD^36^. For eye tracking, in contrast to similar fixation patterns^36^, we found reduced pupil oscillation in participants with ASD during emotion judgment^37^. These results are consistent with studies showing emotion recognition deficits in ASD^38–41^ (see^42–44^ for reviews).
6. Amygdala lesion: We acquired behavioral data from 3 rare patients with focal bilateral amygdala lesions. We have shown an increase in behavioral sensitivity to fear in these patients^12^. This result is in line with other studies on amygdala lesions that have shown altered emotion recognition and judgment^18,45^.

With this dataset, further analysis can be performed to compare different participant groups (e.g., lesion vs. autism) and different neural signals (e.g., EEG vs. fMRI vs. single-neuron). Example usage of this dataset includes a confidence database^46^, computational modeling of single-neuron activity to explain autism behaviors^47^, and multimodal functional connectivity analysis^34^. Along with other multimodal face datasets (e.g.^48^), our current dataset will contribute to a more comprehensive understanding of face perception.

## Methods

The detailed methods have been described in our previous studies^3,4,12,34–37^. Below, we provide an overview of our methods.

### Participants

In this study, we recruited adult participants of both sexes. There was no selection of participants based on age (adults only), sex, race, or ethnicity. The sample size for each experiment was determined empirically based on prior literature. Although we did not perform a formal power analysis to estimate the sample size, given our prior results^3,4,12,34–37^, our data are considered adequate for detecting task effects and for making comparisons using different methodologies.

In the face judgment task with fear-happy morphed emotions, 23 neurotypical participants took part in the EEG experiment, 19 neurotypical participants took part in the fMRI experiment, 24 neurotypical participants took part in the eye tracking experiment, 16 neurosurgical patients (22 sessions) took part in in the single-neuron recording experiment, 18 high-functioning participants with ASD and 15 matched controls (independent of the 24 neurotypical participants for the eye tracking experiment; see more details below) took part in the eye tracking experiment, and 3 rare patients with focal bilateral amygdala lesions took part in the behavioral experiment (see Table 1 for details). Moreover, 16 participants took part in EEG Control Experiment 1 with a speeded response (1a: emotion judgment; 1b: gender judgment; 1c: wealth judgment), 32 participants took part in EEG Control Experiment 2 with modulated ambiguity contexts, 11 participants took part in the EEG Control Experiment 3 with anger-disgust morphed emotions, and 15 participants took part in EEG Control Experiment 4 with (4a) free viewing and (4b) unambiguous decisions (see Table 1 for details).

**Table 1:** Summary of experiments and participants.

Experiment	Stimulus	Task	Type	*n*	Sex (M/F)	Age (mean ± SD years)	CR	Trials	Test location	Language
Main Experiment	Fear-Happy Morph	Fear-Happy Judgment	EEG	23	6/17	22.4 ± 2.17	N	252 trials in 2 blocks	SCNU	Chinese
			fMRI	19	4/15	20.9 ± 2.02	N	168 trials in 2 blocks		
			Eye Tracking	24	8/16	22.3 ± 3.39	Y	252 trials in 3 blocks		
			Neurosurgical	16	11/5	42.3 ± 17.0	12Y/4 N	176 to 440 trials in 2 to 5 blocks	CSMC & HMH	English
			ASD	18	15/3	30.8 ± 7.40	11Y/7 N	252 trials in 3 blocks	CIT	English
			ASD Control	15	11/4	35.1 ± 11.4	Y	252 trials in 3 blocks		
			Amygdala Lesion	3	0/3	36.3 ± 6.35	Y	352 trials in 4 blocks	CIT	English
Control Experiment 1a	Fear-Happy Morph	Speeded Fear-Happy Judgment	EEG	16	5/11	19.6 ± 0.96	Y	280 trials in 2 blocks	SCNU	Chinese
Control Experiment 1b		Speeded Male-Female (Gender) Judgment								
Control Experiment 1c		Speeded Rich-Poor (Wealth) Judgment								
Control Experiment 2	Fear-Happy Morph	Context Modulation		32	17/15	20.6 ± 1.79	N	320 trials in 3 blocks		
Control Experiment 3	Anger-Disgust Morph	Anger-Disgust Judgment		11	2/9	20.6 ± 2.80	N	252 trials in 2 blocks		
Control Experiment 4a	Fear-Happy Morph	Passive Viewing		15	6/9	20.7 ± 1.61	N	168 trials in 1 block		
Control Experiment 4b	Anger-Disgust Morphed Faces and Cat-Dog Morphed Animals	Unambiguous Decisions						252 trials in 2 blocks		

CR: confidence rating. SCNU: South China Normal University. CSMC: Cedars-Sinai Medical Center. HMH: Huntington Memorial Hospital. CIT: California Institute of Technology.

Specifically, all 18 participants with ASD met DSM-V/ICD-10 diagnostic criteria for autism spectrum disorder, and met the cutoff scores for ASD on the Autism Diagnostic Observation Schedule-2 (ADOS-2) revised scoring system for Module 4^49^, and the Autism Diagnostic Interview-Revised (ADI-R)^50,51^ or Social Communication Questionnaire (SCQ)^52^ when an informant was available. The ADOS-2 was scored according to the latest algorithm, and we also derived severity scores for exploratory correlation analyses (social affect (SA): 12.1 ± 4.22 (mean ± SD), restricted and repetitive behavior (RRB): 3.13 ± 1.36, severity score for social affect (CSS SA): 8.00 ± 1.71; severity score for restricted and repetitive behavior (CSS RRB): 7.13 ± 1.36, severity score for social affect plus restricted and repetitive behavior (CSS All): 7.88 ± 1.54). The ASD group had a full-scale IQ (FSIQ) of 105 ± 13.3 (from the Wechsler Abbreviated Scale of Intelligence-2), a mean age of 30.8 ± 7.40 years, a mean Autism Spectrum Quotient (AQ) of 29.3 ± 8.28, a mean SRS-2 Adult Self Report (SRS-A-SR) of 84.6 ± 21.5, and a mean Benton score of 46.1 ± 3.89 (Benton scores 41–54 are in the normal range). ADOS item scores were not available for two participants, so we were unable to utilize the revised scoring system. But these individuals’ original ADOS algorithm scores all met the cutoff scores for ASD. Fifteen neurologically and psychiatrically healthy participants with no family history of ASD (11 male) were recruited as controls. Controls had a comparable FSIQ of 107 ± 8.69 (two-tailed t-test, P = 0.74) and a comparable mean age of 35.1 ± 11.4 years (P = 0.20), but a lower AQ (17.7 ± 4.29, P = 4.62 × 10^−5^) and SRS-A-SR (51.0 ± 30.3, P = 0.0039) as expected. Controls were also matched on sex, race and education.

AP, AM and BG are three patients with selective bilateral amygdala lesions as a result of Urbach-Wiethe disease^53^. AM and BG are monozygotic twins, both with complete destruction of the basolateral amygdala and minor sparing of anterior amygdaloid and ventral cortical amygdaloid parts at a rostral level, as well as lateral and medial parts of the central amygdaloid nucleus and the amygdalohippocampal area at more caudal levels^54^. The details of the neuropsychological assessments of these patients have been described previously^54,55^. The anatomical scans of the lesions are shown in our previous report^12^.

All participants had normal or corrected-to-normal visual acuity. Participants provided written informed consent according to protocols approved by the Institutional Review Board (IRB) of the South China Normal University (SCNU), Huntington Memorial Hospital (HMH), Cedars-Sinai Medical Center (CSMC), and California Institute of Technology (CIT).

### Stimuli

**Fig. 1:**
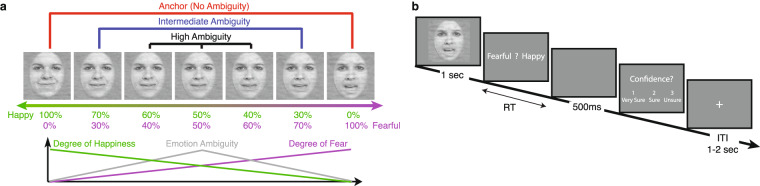
Stimuli and task. (**a**) Sample stimuli of one female identity ranging from 0% fear/100% happy to 100% fear/0% happy. (**b**) Task. A face was presented for 1 s followed by a question asking participants to identify the facial emotion (fearful or happy). After a blank screen of 500 ms, participants were then asked to indicate their confidence in their decision (‘1’ for ‘very sure’, ‘2’ for ‘sure’, or ‘3’ for ‘unsure’). Faces are not shown to scale.

Stimuli were morphed expression continua between exemplars of fearful and happy expressions (see Fig. 1a for examples). Four individuals (two female) were chosen from the STOIC database^56^, a database of face images expressing highly recognizable emotions. The facial expressions were morphed by a computer algorithm; therefore, they are not real faces. For each individual, we selected unambiguous exemplars of fearful and happy expressions as evaluated with normative rating data provided by the database creators. To generate the morphed expression continua for this experiment, we interpolated pixel value and location between fearful exemplar faces and happy exemplar faces using a piece-wise cubic-spline transformation over a Delaunay tessellation of manually selected control points. We created five levels of fear-happy morphs, ranging from 30% fear/70% happy to 70% fear/30% happy in steps of 10%. Low-level image properties were equalized by the SHINE toolbox^57^ (The toolbox features functions for specifying the (rotational average of the) Fourier amplitude spectra, for normalizing and scaling mean luminance and contrast, and for exact histogram specification optimized for perceptual visual quality).

Anger-disgust morphs were created by FaceGen Modeller (http://facegen.com/). Similar to fear-happy morphs, we also selected 4 identities (2 males and 2 females from 3D human face models; two Asian (1 male and 1 female) and two Caucasian), with 2 unambiguous faces and 5 morph levels for each identity. The morphs ranged from 30% anger/70% disgust to 70% anger/30% disgust in steps of 10%.

### Tasks

In the fear-happy judgment task (Fig. 1b), on each trial, a face was presented for 1 second followed by a question prompt asking participants to make the best guess of the facial emotion. Participants reported faces as fearful or happy by pressing a button on the keyboard or response box. After stimulus offset, participants had 2 seconds to respond, otherwise, the trial would be aborted and discarded. Participants were instructed to respond only after stimulus offset. No feedback message was displayed, and the order of faces was completely randomized for each participant. After judging the emotions, participants were asked to indicate their confidence of judgment by pushing the button ‘1’ for ‘very sure’, ‘2’ for ‘sure’, or ‘3’ for ‘unsure’. As with the emotion judgment, participants had 2 seconds to respond before the trial was aborted, and no feedback message was displayed. Confidence rating was omitted for EEG participants, fMRI participants, 7 in-lab ASD participants, and 4 in-lab ASD control participants. An inter-trial-interval (ITI) was jittered randomly with a uniform distribution between 1 and 2 seconds for all participants except 2–8 seconds for fMRI participants.

We conducted the following control experiments for EEG participants, each of which differed from the fear-happy judgment task described above either in stimulus or response. A summary of each experiment is provided in Table 1, and we highlight the distinctions in the descriptions below.

EEG Control Experiment 1 had a speeded response: participants were instructed to respond as quickly as possible. The stimulus stayed on the screen until the button press. Similarly, participants had 2 seconds to respond, otherwise, the trial was aborted and discarded. It is worth noting that in this task, the question prompt asking participants to make the best guess of the facial emotion preceded the stimulus and was presented for 500 ms. In EEG Control Experiment 1a,participants were asked to judge the *emotion* of the fear-happy morphed stimuli. In EEG Control Experiment 1b,participants were asked to judge the *gender* of the stimuli. This task had no ambiguity because all four face models had clearly recognizable genders. In EEG Control Experiment 1c,participants were asked to guess the *wealth* (poor vs. rich) of the face model. This task had the highest ambiguity because whether the face model is poor or rich could not be told without any priors.

EEG Control Experiment 2 modulated the ambiguity contexts: participants only judged anchor faces (i.e., unambiguous faces) in the first and third block (64 trials each) and judged both anchor and morphed faces in the second block (192 trials).

EEG Control Experiment 3 used anger-disgust morphed emotions. The task was the same as the fear-happy judgment task.

EEG Control Experiment 4 modulated judgment responses/decisions. In EEG Control Experiment 4a (free viewing), participants were instructed to freely view the fear-happy morphed faces without pressing any buttons. In EEG Control Experiment 4b (unambiguous decisions), participants were instructed to discriminate whether the stimulus was a human or an animal, using ambiguous anger-disgust morphed faces and cat-dog morphed animals.

### Electroencephalogram (EEG)

Participants were seated comfortably about 1.1 m in front of a computer screen in a dimly lit and electromagnetically shielded room. Experiments were administered on a 19-in. (37.7 × 30.1 cm screen size) IBM LCD display (1280 × 1024 screen resolution). Stimuli were presented using E-prime. EEGs were recorded using a digital AC amplifier from 32 scalp sites with tin electrodes mounted in an elastic cap (NeuroScan4.5) according to the International 10–20 system. EEGs were recorded from the following sites: frontal: FP1, FP2, F7, F3, Fz, F4, F8; frontal-central: FC3, FCz, FC4; central: C3, Cz, C4; central-parietal: CP3, CPz, CP4; parietal: P7, P3, Pz, P4, P8; frontal-temporal-parietal: FT7, TP7, T7, T8, TP8, FT8; and occipital: O1, Oz, O2. The vertical-oculograms (VEOG) were recorded from left supra-orbital and infra-orbital electrodes. The horizontal electro-oculograms (HEOG) were measured from electrodes placed lateral to the outer canthi of the left and right eyes. The ground electrode was placed on the forehead. One reference electrode was placed at the left mastoid and the other at the right mastoid, and all recordings were referenced to the right mastoid. All impedance was maintained below 5ΚΩ. EEG and electro-oculogram (EOG) were amplified using a 0.05–70 Hz band-pass filter and were continuously sampled at 500 Hz for each channel. In Control Experiment 4, EEGs were recorded using a digital AC amplifier from 64 scalp sites.

EEG data were processed using EEGLAB^58^ and in-house MATLAB functions. The continuous EEG data were re-referenced to the average of the left and right external mastoid signals to avoid biasing the data towards one hemisphere^59,60^. The data was filtered using a digital zero-phase shift band-pass filter of 0.5–30 Hz with a slope of 24 dB/octave. Then the continuous EEG data were epoched into 3-s segments (–1000 to 2000 ms relative to stimulus onset; long epoch, for time-frequency analysis) or 1-s segments (–200 to 800 ms relative to stimulus onset; short epoch, for event-related potential [ERP] analysis). The pre-stimulus interval from –400 to –200 ms was used as the baseline for long epochs (consistent with the time-frequency analysis) and the pre-stimulus interval from –200 to 0 ms was used as the baseline for short epochs. The data were baseline corrected by subtracting the average activity during the baseline period.

Trials that had blinks in any part of the segments were excluded using a blink detection tool from ERPlab (http://erpinfo.org/erplab), in which vertical ocular artifacts exceeding a normalized cross-variance threshold of 0.7 were detected during the whole epoch^60,61^. We rejected these trials because blinks might not only alter the sensory input of that trial, but also contaminate the EEG signals, especially the signals from the frontal channels. Although we did not explicitly instruct participants not to explore the face during face presentation, the fixation cross preceding the stimulus could reduce eye movements and artifacts related to eye movements. Epochs with saccadic eye movements were detected and discarded using a step-like artifact-detection function, in which horizontal ocular artifacts exceeding 70 μV in amplitude were detected during the entire epoch with 200 ms moving window and 50 ms increment steps. This function is suitable to detect saccadic eye movements that typically consist of sudden, step-like changes in voltage^61^. Remaining artifacts were further detected using a moving-window peak-to-peak artifact-detection method on specific midline electrodes. Epochs were excluded if the peak-to-peak voltage (the difference between the largest and smallest values) exceeded a threshold of 100 μV. Bad channels were interpolated using the average voltage from their surrounding electrodes. Notably, we repeated our artifact rejection using independent component analysis (ICA) and we derived qualitatively the same results.

Within each participant, mean waveform of each morph/ambiguity level was computed, time-locked to the onset of the stimulus. Single-participant mean waveforms were subsequently averaged to obtain group-level mean waveforms. Here, we measured the LPP (entire waveform) based on the time window of 400 to 700 ms after stimulus onset at the parietal-central (Pz) electrode^62^. Importantly, the scalp topography of the difference waveform between high ambiguity and unambiguous stimuli showed the most pronounced difference at Pz in this time window^3^.

### Functional magnetic resonance imaging (fMRI)

MRI scanning was conducted at the SCNU on a 3-Tesla Tim Trio Magnetic Resonance Imaging scanner (Siemens, Germany) using a standard 12-channel head-coil system. Stimuli were presented to the participants on a back-projection screen using MATLAB with the Psychophysics Toolbox^63^. Stimuli were presented using a mirror attached to the MRI head coil. Whole-brain data were acquired with echo planar T2*-weighted imaging (EPI), sensitive to blood-oxygen-level dependent (BOLD) signal contrast (31 oblique axial slices, 3 mm-thicknesses; TR** = **2000 ms; TE = 30 ms; flip angle = 90°; FOV = 224 mm; voxel size: 3 × 3 × 3 mm). T1 weighted structural images were acquired at a resolution of 1 × 1 × 1 mm.

Neuroimaging data were preprocessed and analyzed using SPM12 (www.fil.ion.ucl.ac.uk/spm/). The first 4 volumes were discarded to allow the MR signal to reach steady-state equilibrium. EPI images were sinc interpolated in time to correct for slice-timing differences and realigned to the first scan by rigid-body transformations to correct for head movements. Utilizing linear and nonlinear transformations and smoothing with a Gaussian kernel of full-width-half maximum 6 mm, EPI and structural images were coregistered to the T1 MNI 152 template (Montreal Neurological Institute, International Consortium for Brain Mapping). Global changes were removed by high-pass temporal filtering with a cutoff of 128 s to remove low-frequency drifts in signal.

We used an event-related design. In the general linear model (GLM) design matrix, for every participant we estimated a GLM with autoregressive order 1 [AR(1)] and the following regressors (R): R1 at face presentation; R2 at face presentation modulated by fear levels: 100%, 70%, 60%, 50%, 40%, 30%, 0%; R3 at face presentation modulated by ambiguity levels: unambiguous, 30%/70% morph (intermediate ambiguity), 40–60% morph (high ambiguity); and R4 at fixation presentation. For all GLM analyses, six head-motion regressors based on the SPM’s realignment estimation routine were added to the model (aligned to the first slice of each scan). Multiple linear regressions were then run to generate parameter estimates for each regressor for every voxel. The contrast (difference in beta values) images of the first-level analysis were entered into one-sample *t*-tests for the second-level group analysis conducted with a random-effects statistical model^64^.

### Single-neuron recordings

We recorded bilaterally from implanted depth electrodes in the amygdala and dmPFC (including dorsal anterior cingulate cortex [dACC] and pre-supplementary motor area [pre-SMA]) from patients with pharmacologically intractable epilepsy. Target locations were verified using post implantation structural MRIs. At each site, we recorded from eight 40 μm microwires inserted into a clinical electrode as described previously^65^. Bipolar wide-band recordings (0.1–9 kHz), using one of the eight microwires as reference, were sampled at 32 kHz and stored continuously for off-line analysis with a Neuralynx system (Digital Cheetah; Neuralynx, Inc.). The raw signal was filtered with a zero-phase lag 300-3 kHz bandpass filter and spikes were sorted using a semiautomatic template matching algorithm^66^. Units were carefully isolated and spike sorting quality were assessed quantitatively.

To analyze the response of individual neurons, we only considered single units with an average firing rate of at least 0.2 Hz (entire task). Trials were aligned to face onset, and the baseline firing rate was calculated in a 1-s interval of blank screen right before face onset. To select neurons that showed a significant trial-by-trial correlation with the level of emotion ambiguity, we quantified the response of each neuron based on the number of spikes in a 1.5-s window starting 250 ms after stimulus onset. Peristimulus time histograms (PSTH) were computed by counting spikes across trials in consecutive 250 ms bins. Comparisons between morph/ambiguity levels in the PSTH were made using a one-way ANOVA at P < 0.05 and Bonferroni-corrected for multiple comparisons across bins.

### Eye tracking

In the eye tracking experiment with neurotypical participants conducted at the SCNU (Table 1), we used two eye tracking systems. Fourteen participants were recorded with a head-supported noninvasive infrared EyeLink 1000 System (SR Research). The stimuli were presented using MATLAB with the Psychophysics Toolbox. One of the eyes was tracked at 1000 Hz. Participants were seated at a distance of 60 cm in front of a computer screen in a dimly lit, sound-attenuated room. The experiment was administered on a 20-inch (40 × 30-cm screen size) Lenovo CRT display (1024 × 768 screen resolution). The eye tracker was calibrated with the built-in 9-point grid method at the beginning of each block. Fixation extraction was conducted using software supplied with the EyeLink eye tracking system. Saccade detection required a deflection of >0.1°, with a minimum velocity of 30°/s and a minimum acceleration of 8000°/s^2^. Fixations were defined as the complement of a saccade, i.e., periods without saccades, and the fixation locations were determined using the EyeLink event parser. Ten healthy participants were recorded using a remote noninvasive infrared Tobii T120 system which recorded binocular gaze at 120 Hz. The stimuli were presented using Matlab with the Psychophysics Toolbox. The Tobii visualization software (Tobii Studio 2.2) was used to record eye movements and perform gaze analysis. Fixations were detected by Tobii Fixation Filter implemented in Tobii Studio. The Tobii Fixation Filter is a classification algorithm proposed by Olsson^67^ and detects quick changes in the gaze point using a sliding window averaging method. The velocity threshold was set to 35 pixels/sample, and the distance threshold was set to 35 pixels in our study.

In the eye tracking experiment conducted at the CIT focusing on studying ASD (Table 1), 18 participants with ASD and 15 matched controls were recorded using a non-invasive infrared remote Tobii TX300 system that enables recording of eye movements and pupil size as well as detection of visual fixations (sliding window averaging method; velocity threshold = 35 pixels/sample). We excluded all trials where only one eye was tracked, which indicated head turning that could introduce error from ambient light exposure. Blinks were detected by Tobii Studio and were labeled as missing data, so we excluded all blinks from analysis.

To quantitatively compare the fixation properties within certain parts of the face, we defined three regions of interest (ROIs): eyes, mouth, and center. Each ROI is a rectangle, and the eye and mouth ROI have the same size. To compute fixation density maps, fixation locations were smoothed with a 40-pixel 2D Gaussian kernel with a standard deviation of 10 pixels. The fixation density map indicates the probability of fixating a given location (in arbitrary units), which was calculated based on the number and duration of fixations and ensured an equal contribution from each participant and statistical independence between participants. The average fixation density within the ROIs was calculated for each participant and for each morph level during the entire stimulus period. Statistical comparisons were then performed to compare whether the mean fixation density, the total fixation duration, the mean fixation duration, the percentage of the number of fixations, and the latency of the first fixation falling into an ROI differed between fear/ambiguity levels, for each ROI.

## Data Records

The data and code^68^ were sorted out in separate directories based on the data formats and experiments. A detailed description of the variables and usage of data and code can be found in the corresponding ‘README.docx’ file in each data directory.

### Stimuli

The stimuli used in this study are stored in the ‘/01 Stimuli/’ directory. Additionally, an independent group of 10 participants from the SCNU provided valence and arousal ratings for the fear-happy stimuli. Each face was rated five times on a scale from 1 to 10. For valence, participants were asked to rate how pleasant the displayed emotion of the face was, with 1 representing ‘very unpleasant’ and 10 representing ‘very pleasant’. For arousal/intensity, participants were asked to rate how intense the displayed emotion of the face was, with 1 representing ‘very mild/calm’ and 10 representing ‘very intense/excited’. The original behavioral data for each participant are stored in the corresponding ‘.DAT’ files, where ‘v_’ represents valence ratings and ‘a_’ represents arousal ratings. Additionally, a group summary of the ratings is stored in the file ‘FaceRatingSummary.xlsx’.

### Behavioral data

Behavioral data from all participants (EEG, fMRI, eye tracking, ASD, ASD matched controls, single-neuron, lesion) are stored in the file ‘behaviorData.mat’ in the ‘/02 Behavioral Data and Code/’ directory. Each element of the structure array corresponds to a specific participant. The code for the seven levels of fearfulness (ranging from 1 for the most fearful to 7 for the happiest) is stored in the field ‘codeL’. The code for the face identity is stored in the field ‘codeI’. The button press responses, where 1 represents a fearful response and 2 represents a happy response, are stored in the field ‘vResp’. Response times are recorded and stored in the field ‘RT’. Confidence ratings are stored in the field ‘vCR’. Response times for confidence ratings are stored in the field ‘RT_CR’. It is worth noting that EEG and fMRI participants as well as a subset of ASD, ASD control, and single-neuron participants did not provide confidence ratings.

### EEG data

The preprocessed EEG data and code are stored in the ‘/03 EEG Data and Code/’ directory. The EEG data after artifact rejection using long epochs (–1000 to 2000 ms relative to stimulus onset) are stored in the file ‘Data_FearHappy_LongEpoch.zip’. The long epochs are better suited for time-frequency analysis. Additionally, the EEG data after artifact rejection using short epochs (–200 to 800 ms relative to stimulus onset) are stored in the file ‘Data_FearHappy_ShortEpoch.zip’. The short epochs are better suited for ERP analysis. Here, we focused on the LPP based on the time window of 400 to 700 ms after stimulus onset at the parietal-central (Pz) electrode^62^ because the scalp topography of the difference waveform between high ambiguity and unambiguous stimuli showed the most pronounced difference at Pz in this time window^3^. The LPP waveforms for three levels of ambiguity can be obtained by running the script ‘LPP_plots_3levels_ambiguity_longepoch.m’ for the long-epoch data or ‘LPP_plots_3levels_ambiguity_shortepoch.m’ for the short-epoch data. The mean and peak amplitudes of the LPP are stored in the files ‘LPP_long.mat’ and ‘LPP_short.mat’, respectively. The mean and peak amplitudes for three levels of ambiguity can be plotted using the scripts ‘LPP_bars_longepoch.m’ and ‘LPP_bars_shortepoch.m’, which load the files ‘LPP_long.mat’ and ‘LPP_short.mat’, respectively. In addition, similar analyses can be performed for the N170 component using the script ‘N170_plots_3levels_ambiguity_shortepoch.m’.

The data and code for the control experiments are stored in a similar format in the same directory. Each control experiment is stored in a separate zip file.

### fMRI data

The preprocessed fMRI data are stored in the ‘/04 fMRI Data and Code/’ directory in two files: ‘sub1-10.zip’ and ‘sub11-19.zip’. The file ‘onsets.zip’ contains all the behavioral data and code necessary for extracting the stimulus onsets for the parametric design. The script ‘step1_firstlevel_model_face_fear_ambiguity3.m’ generates the first-level parametric model for each participant. The script ‘step2_firstlevel_contrasts_face_para3.m’ generates the first-level contrasts for each participant. The script ‘step3_secondlevel_model_face_para3.m’ generates the second-level (group average) contrasts. The file ‘rfx_face_emotion_para3.zip’ contains all the group-averaged contrasts. These steps are performed using SPM12 in MATLAB. The file ‘Brain Activation.docx’ documents all the coordinates of the activated brain regions.

### Single-neuron data

Single-neuron data from the amygdala and MFC are stored in the file ‘FiringRate_Amygdala.mat’ and ‘FiringRate_MFC.mat’, respectively, in the ‘/05 Single-neuron Recordings Data and Code/’ directory. Firing rates estimated within different time windows for each neuron are stored in the variable ‘FR’. For each neuron, the timestamps (in μs), session ID, channel ID, cluster ID, and recording brain area are stored in the variables ‘timestampsOfCellAll’, ‘vCell’, ‘vCh’, ‘vClusterID’, and ‘areaCell’, respectively (note that these variables were all matched). “sessions” contains the session identifiers corresponding to the index in ‘vCell’. The variable ‘beh’ stores the behavior for each recording session (in the same format as described above in the section ‘Behavioral data’), and the variable ‘sessions’ stores detailed participant information. The file ‘plotRastersEachCell.m’ provides a demonstration of a raster plot for a given neuron. The file ‘plotGroupPSTH.m’ provides a demonstration of the group PSTH and a dot plot for group average.

### Eye tracking data

Eye tracking data are stored in the file ‘/06 Eye-tracking Data and Code/ET_Data_All.mat’. The eye tracking data for each trial were aligned to the image coordinates and then were extracted and stored in a matrix format in the variable ‘EM’. In each matrix, each row refers to a single fixation and the following saccade and the columns (28 in total) refer to the following attributes: 1—trial index, 2—fixation index, 3—fixation coordinate in horizontal direction (aligned to image coordinates), 4—fixation coordinate in vertical direction, 5—fixation start time (in ms, relative to image onset), 6—fixation end time, 7—type of stimuli (here we only included type ‘1’ for fear-happy morphs), 8—ROI that the fixation falls in, 9—the serial order of fixation in each ROI, 10—empty (not used), 11—empty (not used), 12—fixation duration, 13—saccade index, 14—saccade starting position in horizontal direction, 15—saccade starting position in vertical direction, 16—saccade ending position in horizontal direction, 17—saccade ending position in vertical direction, 18—saccade starting time, 19—saccade ending time, 20—saccade duration, 21—saccade distance (in degrees of visual angle), 22—saccade peak velocity (in visual degrees per second), 23—saccade starting ROI (1: eyes, 2: mouth, 3: nose, 0: other), 24—saccade ending ROI, 25—serial order of saccade with starting position in this ROI (e.g., ‘2’ means the second saccade in this trial with the starting position in this ROI), 26—serial order of saccade with ending position in this ROI, and 27—saccade direction (e.g., a number ‘12’ denotes saccade from the eyes to the mouth; note that digit 4 rather than 0 is used here to denote other parts). Note that data recorded using the Tobii eye tracker do not have the saccade information available.

Furthermore, the variable ‘vSub’ denotes the participant category, the variable ‘beh’ stores the behavior (in the same format as described above in the section ‘Behavioral data’), and the variable ‘sessions’ stores detailed participant information. It is important to note that all of these variables correspond to ‘EM’. Additionally, the variables ‘ROI_E’, ‘ROI_M’, and ‘ROI_C’ denote the ROI coordinates for the eyes, mouth, and center, respectively. The file ‘analyzeFixationDensityDemo’ plots fixation density maps for each participant group and the file ‘analyzeFixationAttributesDemo’ plots the percentage of the number of fixations, total fixation duration, mean fixation duration, and first fixation latency for each ROI. The file ‘analyzeFixationAttributesDemo’ also demonstrates comparisons between participant groups, across morph/stimulus levels, and across ambiguity levels.

## Technical Validation

### Behavior

We first assessed the quality of our behavioral data and demonstrated that smooth psychometric curves could be derived from participants’ choices. Additionally, we showed that reaction time (RT) and participants’ confidence in their decisions varied as expected based on the stimulus.

**Fig. 2:**
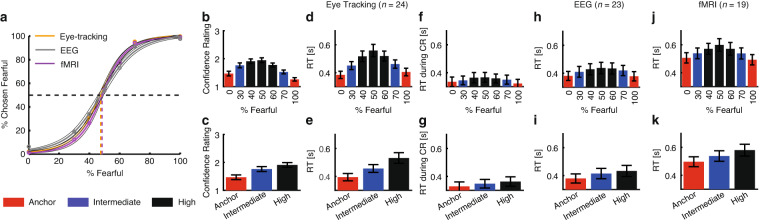
Behavioral results. (**a**) Group average of psychometric curves. We fitted a logistic function to obtain smooth psychometric curves^12^. Shaded area denotes ± SEM across participants. (**b**) Confidence rating (CR) as a function of fearful level. (**c**) Confidence rating as a function of ambiguity level. (**d,h,j**) Reaction time (RT) for the fear/happy decision as a function of fearful level. (**e,i,k**) RT as a function of ambiguity level. (**f**) RT during confidence rating as a function of fearful level. (**g**) RT during confidence rating as a function of ambiguity level. Error bars denote ± SEM across participants.

For each participant, we quantified behavior as the proportion of trials identified as fearful as a function of morph level (Fig. 2a). We found a monotonically increasing relationship between the likelihood of identifying a face as fearful and the fearfulness in the morphed face for all participant groups (Fig. 2a; note that here we exemplified the results using EEG, fMRI, and eye tracking participants, but we derived similar results for all other participant groups; also note that participants with ASD had flatter psychometric curves^36^ and amygdala lesion patients had shifted psychometric curves^12^).

We found that RT was faster for anchor faces (i.e., unambiguous faces) compared to ambiguous faces for each participant group (one-way repeated-measure ANOVA of morph levels; eye tracking [Fig. 2d]: F(6,138) = 19.0, P < 0.001, η_p_^2^ = 0.45; EEG [Fig. 2h]: F(6,132) = 5.14, P < 0.001, η_p_^2^ = 0.19; fMRI [Fig. 2j]: F(6,102) = 8.71, P < 0.001, η_p_^2^ = 0.33). When grouping all trials into three levels of ambiguity, each group of participants showed the shortest RT for anchor faces (no ambiguity) and the longest RT for high ambiguity faces (one-way repeated-measure ANOVA of ambiguity level; eye tracking [Fig. 2e]: F(2,46) = 31.7, P < 0.001, η_p_^2^ = 0.58; EEG [Fig. 2i]: F(2,44) = 11.0, P < 0.001, η_p_^2^ = 0.33; fMRI [Fig. 2k]: F(2,36) = 11.3, P < 0.001, η_p_^2^ = 0.39).

Eye tracking participants reported their confidence in their decisions after reporting a face as fearful or happy. They reported significantly higher levels of confidence for anchor faces compared to ambiguous faces (one-way repeated-measure ANOVA of morph levels: F(6,138) = 42.0, P < 0.001, η_p_^2^ = 0.65; Fig. 2b; one-way repeated-measure ANOVA of ambiguity levels: F(2,46) = 72.6, P < 0.001, η_p_^2^ = 0.76; Fig. 2c). RT during confidence rating slightly varied as a function of ambiguity levels (one-way repeated-measure ANOVA of morph levels: F(6,138) = 2.17, P = 0.094, η_p_^2^ = 0.086; Fig. 2f; one-way repeated-measure ANOVA of ambiguity levels: F(2,46) = 3.72, P = 0.046, η_p_^2^ = 0.139; Fig. 2g).

### EEG

We next assessed the quality of our EEG data for each experiment. Specifically, we validated across experiments that the LPP served as an important index for decision-making on emotional ambiguity.

1. In the fear-happy judgment task (Fig. 3a), we found that the LPP showed a parametric relationship with the degree of ambiguity in the stimuli. Our results were further confirmed by the mean LPP amplitude (one-way repeated-measure ANOVA of ambiguity levels, F(2,44) = 11.1, P = 1.27 × 10^−4^, η_p_^2^ = 0.34), and post-hoc t-tests revealed a significant difference between anchor (5.56 ± 1.87 µv; mean ± SD) and intermediate ambiguity (4.45 ± 1.36 µv; paired two-tailed t-test, t(22) = 3.17, P = 0.004, Cohen’s d = 0.66), and a marginal difference between intermediate and high ambiguity (4.05 ± 1.42 µv; t(22) = 1.73, P = 0.098, d = 0.36). In addition to the LPP, we also observed an N170 component in electrodes TP7 and TP8 in response to emotional facial images. However, the N170 was not sensitive to varying degrees of emotion ambiguity (F(2,44) = 0.406, P = 0.669, η_p_^2^ = 0.018) or valence (F(2,44) = 0.659, P = 0.522, η_p_^2^ = 0.029).

**Fig. 3:**
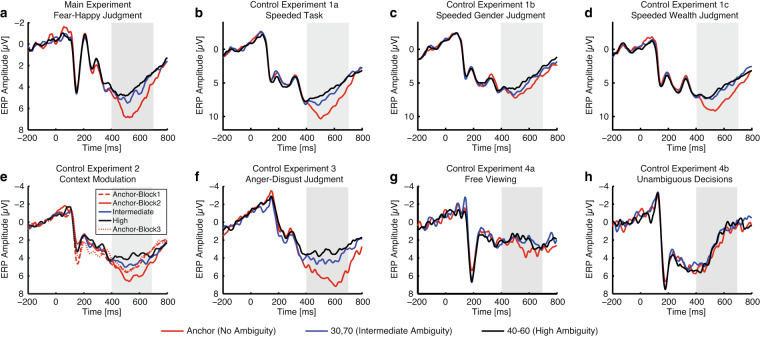
EEG results. We plotted the event-related potential (ERP) at the electrode Pz as a function of ambiguity levels. Gray shaded area denotes the LPP. (**a**) Fear-happy judgment task. (**b**) Control Experiment 1a: speeded task. Similar LPP results were derived in the speeded task. (**c**) Control Experiment 1b: speeded task with gender judgment. The LPP could still differentiate levels of stimulus ambiguity, but to a lesser extent. (**d**) Control Experiment 1c: speeded task with wealth judgment. The LPP could differentiate levels of stimulus ambiguity. (**e**) Control Experiment 2: context modulation. The LPP was not only modulated by ambiguity levels, but also by the context of ambiguous stimuli. (**f**) Control Experiment 3: anger-disgust judgment task. Anger-disgust morphed faces elicited similar LPP signals in response to ambiguity. (**g**) Control Experiment 4a: free viewing of the stimuli. The LPP was abolished when participants freely viewed the faces without judging emotions. (**h**) Control Experiment 4b: judgment with unambiguous decisions. The LPP was abolished when participants judged whether the stimulus was a human face or an animal, an unambiguous aspect of the stimuli.
2. In Control Experiment 1a (a fully speeded version of the task; Fig. 3b), we replicated the results found in the fear-happy judgment task: the LPP amplitude can index the level of emotion ambiguity. Our results were confirmed by the mean LPP amplitude (F(2,30) = 48.0, P = 4.48 × 10^−10^, η_p_^2^ = 0.76).
3. In Control Experiment 1b (speeded gender judgment task; Fig. 3c), although the LPP could still differentiate levels of stimulus ambiguity (F(2,30) = 8.48, P = 0.0012, η_p_^2^ = 0.36), the coding of stimulus ambiguity was relatively weaker compared with the emotion judgment task.
4. In Control Experiment 1c (speeded wealth judgment task; Fig. 3d), we found that the LPP could differentiate levels of stimulus ambiguity (F(2,30) = 33.3, P = 2.41 × 10^−8^, η_p_^2^ = 0.69).
5. In Control Experiment 2 (context modulation; Fig. 3e), we designed a 3-block experiment: in the first block, participants only judged unambiguous faces; in the second block, participants judged both unambiguous and morphed faces; and in the third block, participants again only judged unambiguous faces. In the second block, we still observed a distinct LPP that differentiated the levels of ambiguity. However, when there were no ambiguous trials, the LPP associated with unambiguous faces was much weaker in the first and third blocks. This suggests that the context of ambiguous stimuli influenced the modulation of the LPP. Analyzing the mean LPP amplitude through post-hoc t-tests revealed a significant difference between unambiguous faces in the first and second blocks (paired two-tailed t-test, t(31) = 3.01, P = 0.0052, d = 0.55), as well as between the second and third blocks (t(31) = 3.05, P = 0.0046, d = 0.55). However, there was no significant difference in unambiguous faces between the first and third blocks (t(31) = 0.39, P = 0.70, d = 0.07), indicating that the influence of the ambiguous stimuli in the second block was transient and did not carry over into the third block. Notably, the mean LPP amplitude in the second block varied according to the different levels of ambiguity (F(2,62) = 21.27, P = 9.22 × 10^−8^, η_p_^2^ = 0.407). Therefore, the LPP was influenced not only by the levels of ambiguity but also by the context of ambiguous stimuli.
6. In Control Experiment 3 (anger-disgust morphed emotions; Fig. 3f), we obtained similar LPP results as in the fear-happy judgment task. This observation was confirmed by the mean LPP amplitude (F(2,20) = 10.59, P = 0.001, η_p_^2^ = 0.51).
7. In Control Experiment 4a (passive viewing; Fig. 3g), participants passively viewed the faces without making any decisions about facial emotions. We even found no LPP, and confirmed no significant difference in the LPP interval for ambiguity levels using the mean amplitude (F(2,28) = 0.63, P = 0.54, η_p_^2^ = 0.04).
8. In Control Experiment 4b (unambiguous decisions; Fig. 3h), participants judged whether the stimulus was a human face or an animal. Although both the face and animal stimuli were ambiguous, the decisions/judgments on animal or human face are certain. Again, for both types of stimuli, we found no LPP, and confirmed no significant difference in the LPP interval for ambiguity levels using the mean amplitude (F(2,28) = 0.45, P = 0.64, η_p_^2^ = 0.03).

It is worth noting that in some experiments, there was ramping activity in the baseline, likely due to anticipation of the stimulus. However, given the time window of the LPP, such ramping activity in the baseline was not likely to impact the interpretation of our results. In particular, we observed no significant difference across conditions in the baseline.

### fMRI

**Fig. 4:**
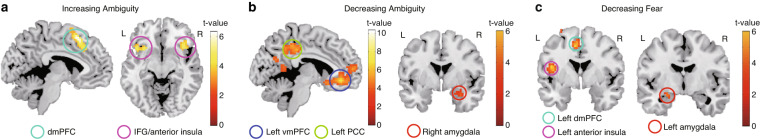
fMRI results. (**a**) Increasing ambiguity was correlated with increasing BOLD activity in the bilateral dorsomedial prefrontal cortex (dmPFC) and inferior frontal gyrus (IFG)/anterior insula. The generated statistical parametric map was superimposed on anatomical sections of the standardized MNI T1-weighted brain template. L: left. R: right. (**b**) Decreasing ambiguity was correlated with increasing BOLD activity in the right amygdala, left ventromedial prefrontal cortex (vmPFC), and posterior cingulate cortex (PCC). (**c**) Decreasing fear was correlated with increasing BOLD activity in the left amygdala, left dmPFC, and left insula.

We next validated the fMRI data. We demonstrated the following BOLD-fMRI activations: (1) a significant increase of BOLD signal in the bilateral inferior frontal gyrus (IFG)/anterior insula and dorsal medial prefrontal cortex (dmPFC)/dorsal anterior cingulate cortex (dACC) as a function of increasing emotion ambiguity (Fig. 4a), (2) a significant increase of BOLD signal in the right amygdala, left ventral ACC (vACC), posterior cingulate cortex (PCC), dorsolateral prefrontal cortex (dlPFC), inferior parietal lobule (IPL), and right postcentral gyrus as a function of decreasing emotion ambiguity (Fig. 4b), and (3) a significant increase of BOLD signal in the left amygdala, dmPFC, and insula as a function of decreasing fear intensity (Fig. 4c). Together, our data revealed a network of brain regions that encoded emotion ambiguity and fear intensity.

### Single-neuron recordings

**Fig. 5:**
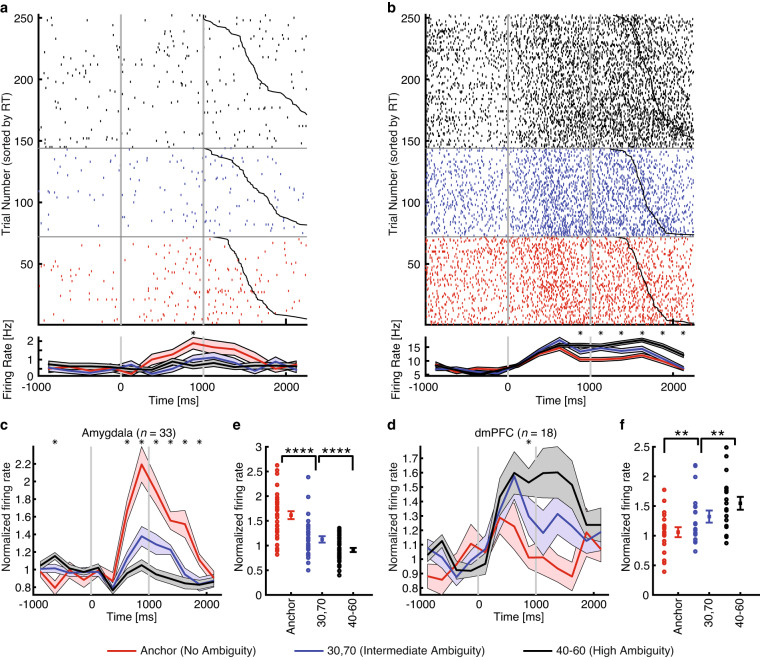
Single-neuron results. (**a**) An example amygdala neuron that fire most to unambiguous faces and least to the most ambiguous faces (linear regression: P < 0.05). (**b**) An example dorsomedial prefrontal cortex (dmPFC) neuron that fire most to the most ambiguous faces and least to unambiguous faces (linear regression: P < 0.05). Raster (top) and PSTH (bottom) are color coded according to ambiguity levels as indicated. Trials are aligned to face stimulus onset (left gray bar, fixed 1 s duration) and sorted by reaction time (black line). PSTH bin size is 250 ms. Shaded area and error bars denote ± SEM across trials. Asterisk indicates a significant difference between the conditions in that bin (P < 0.05, one-way ANOVA, Bonferroni-corrected). (**c,** **d**) Average normalized firing rate of ambiguity-coding neurons. Asterisk indicates a significant difference between the conditions in that bin (P < 0.05, one-way ANOVA, Bonferroni-corrected). (**e,** **f**) Mean normalized firing rate at ambiguity level. Normalized firing rate for each unit (left) and mean ± SEM across units (right) are shown at each ambiguity level. Mean firing rate was calculated in a time window 250 to 1750 ms after stimulus onset (the same time window as neuron selections). Asterisks indicate a significant difference between conditions using paired two-tailed *t*-test. **P < 0.01 and ****P < 0.0001. (**c,** **e**) Neurons in the amygdala that increased their firing rate for the least ambiguous faces (*n* = 33). (**d,** **f**) Neurons in the dmPFC that increased their firing rate for the most ambiguous faces (*n* = 18).

We recorded from 321 neurons in the amygdala (21 sessions) and 236 neurons in the dmPFC (15 sessions; overall firing rate greater than 0.2 Hz). To validate our data, we demonstrated that the responses of amygdala and dmPFC neurons were modulated by the level of emotion ambiguity. Specifically, we used a linear regression to identify neurons whose firing rate correlated trial-by-trial with three levels of emotion ambiguity. We found 36 amygdala neurons (11.2%; binomial P = 2.58 × 10^−6^; see Fig. 5a for an example and Fig. 5c,e for group result) and 29 dmPFC neurons (12.3%; binomial P = 3.09 × 10^−6^; see Fig. 5b for an example and Fig. 5d,f for group result) that showed a significant trial-by-trial correlation. Together, our data revealed neurons in the amygdala and dmPFC that encoded levels of emotion ambiguity.

### Eye tracking

**Fig. 6:**
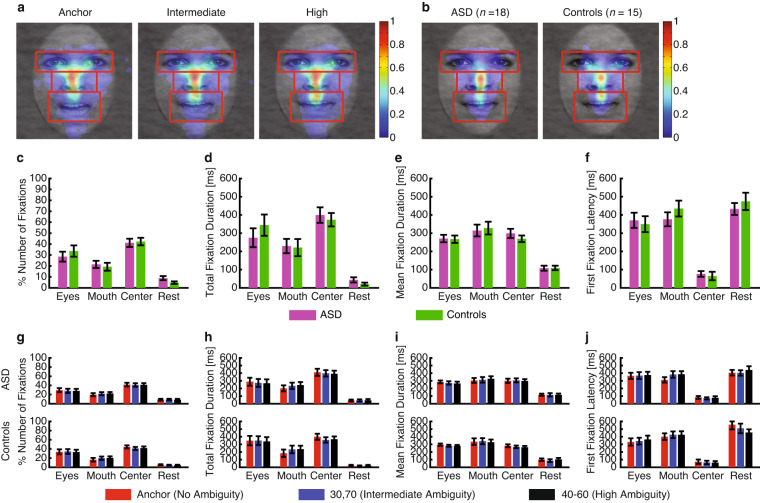
Eye tracking results. (**a**) Fixation density maps to quantify eye movements for each ambiguity level. (**b**) Fixation density maps to quantify eye movements for participants with autism spectrum disorder (ASD) and matched controls. Each map shows the probability of fixating a given location within a 1-s period after stimulus onset. The ROIs (eye, mouth, center) used for analysis are shown in red (not shown to participants). (**c**–**f**) Comparison between participants with ASD and matched controls. (**g**–**j**) Comparison across ambiguity levels for each participant group (upper row: ASD; lower row: controls). (**c,g**) Percentage of the number of fixations in each ROI. (**d,h**) Total fixation duration in each ROI. (**e,i**) Mean fixation duration in each ROI. (**f,j**) Latency of the first fixation onto each ROI. Error bars denote ± SEM across participants.

We lastly validated the eye tracking data. We demonstrated indistinguishable fixation densities across ambiguity levels (Fig. 6a), in which participants were equally likely to fixate the eye (one-way repeated-measure ANOVA of ambiguity levels, P = 0.91), mouth (P = 0.62), and center ROIs (P = 0.95), suggesting that participants viewed faces similarly regardless of the ambiguity in faces. Furthermore, we found that people with ASD had a similar fixation pattern as matched controls (Fig. 6b). These results were further confirmed by a similar percentage of the number of fixations (Fig. 6c), total fixation duration (Fig. 6d), mean fixation duration (Fig. 6e), and first fixation latency (Fig. 6f) for each ROI. These fixation attributes were similar across ambiguity levels for each ROI and participant group (Fig. 6g–j).

## Usage Notes

The code for analyzing each modality of the data is located in the corresponding individual directory, along with a detailed “README” file. By running the scripts in a designated order (described in the each “README” file), the same figures stored in the ‘output figures’ folder can be obtained.

## Data Availability

The source code is included as part of the dataset^68^. All code is implemented using MATLAB (MathWorks Inc.)
